# Long-Term Prognosis of Patients With Transient Ischemic Attack or Stroke and Symptomatic Vascular Disease in Multiple Arterial Beds

**DOI:** 10.1161/STROKEAHA.118.020913

**Published:** 2018-06-07

**Authors:** Mirjam R. Heldner, Linxin Li, Nicola G. Lovett, Magdalena M. Kubiak, Shane Lyons, Peter M. Rothwell

**Affiliations:** From the Centre for Prevention of Stroke and Dementia, Nuffield Department of Clinical Neurosciences, University of Oxford, United Kingdom.

**Keywords:** atherosclerosis, coronary artery disease, peripheral vascular disease, prognosis, stroke

## Abstract

**Background and Purpose—:**

Cerebrovascular, coronary, and peripheral vascular disease have common underlying arterial pathology and risk factors, but the clinical significance of multiple-territory disease in patients with transient ischemic attack (TIA)/ischemic stroke is unclear, particularly whether the number of clinically affected territories still predicts long-term outcome on current standard secondary prevention therapies.

**Methods—:**

In a population-based study of 92 728 individuals in Oxfordshire, United Kingdom (Oxford Vascular Study), we studied patients presenting with TIA/ischemic stroke (2002–2014) in relation to the number of other vascular beds (coronary, peripheral) affected by symptomatic (current or previous) disease. We compared the risk factor profile and long-term prognosis in patients with single- versus multiple-territory disease.

**Results—:**

Among 2554 patients with 10 679 patient-years of follow-up, 1842 (72.1%) had single- (TIA/stroke only), 608 (23.8%) double-, and 104 (4.1%) triple-territory symptomatic vascular disease. The number of affected vascular beds increased with the number of atherosclerotic risk factors (*P*_trend_<0.0001). Compared with patients with TIA/stroke only, those with multiple-territory disease had more hypertension (age/sex-adjusted odds ratio [OR], 3.43; 95% confidence interval [CI], 2.76–4.27; *P*<0.0001), diabetes mellitus (OR, 2.89; 95% CI, 2.27–3.66; *P*<0.0001), hypercholesterolemia (OR, 4.67; 95% CI, 3.85–5.66; *P*<0.0001), and current or previous smoking (OR, 1.52; 95% CI, 1.26–1.84; *P*<0.0001). Triple-territory disease was particularly strongly associated with hypercholesterolemia (OR, 6.80; 95% CI, 4.39–10.53; *P*<0.0001). Despite more intensive secondary prevention in patients with multiple-territory disease, the 5-year risk of vascular death increased steeply with the number of territories affected (17.2% versus 30.0% versus 42.9%; *P*<0.0001). Compared with patients with single-territory, patients with multiple-territory disease also had higher postacute long-term risks (90 days to 10 years) of recurrent ischemic stroke (age/sex-adjusted hazard ratio, 1.38; 95% CI, 1.04–1.81; *P*=0.02) and nonstroke acute vascular events (hazard ratio, 3.06; 95% CI, 2.23–4.20; *P*<0.0001).

**Conclusions—:**

Number of affected vascular beds appeared to be a simple clinical rule in identifying TIA/ischemic stroke patients who are at high long-term risk of nonstroke vascular events and vascular death.

## Abstract 2

Supplemental Digital Content is available in the text.

Atherosclerotic vascular disease is a chronic disease of the arterial wall of different vascular territories, causing cerebrovascular, coronary, peripheral, and aortic vascular disease. It is the leading cause of death and morbidity worldwide.^1^ Vascular risk factors, such as hypertension and hypercholesterolemia are important drivers of atherosclerosis^2–5^ and blood pressure lowering, lipid-lowering, and antiplatelet treatment are effective in reducing acute vascular events both in primary and secondary prevention settings.^6–9^ Recent randomized trials showed that novel anti-inflammatory and lipid-lowering therapies reduced risk of recurrent cardiovascular events in patients with cardiovascular disease on current standard secondary prevention treatment.^10–12^ However, these new agents are expensive and are unlikely to be cost-effective in patients at low vascular risk.

Patients in the secondary prevention setting with atherosclerotic disease affecting 2 or more vascular beds seem to be at high risk for future vascular events.^13,14^ However, previous studies were hospital-based, had a relatively short follow-up, and did not focus specifically on patients with transient ischemic attack (TIA) and stroke. To assess whether TIA/ischemic stroke patients with disease in other vascular beds were at particularly high risk of future vascular events as previously suggested,^13,14^ we studied patients presenting with TIA or ischemic stroke in relation to the number of other vascular beds (coronary, peripheral) affected by symptomatic disease to determine long-term prognosis on current standard secondary prevention in a population-based study. We hypothesized that the number of affected vascular beds could be used as a simple clinical rule in identifying patients who are at high risk of recurrent vascular events.

## Methods

Requests for access to data from OXVASC will be considered by the corresponding author.

We studied consecutive patients with a first-in-the-study-period TIA or ischemic stroke in OXVASC (Oxford Vascular Study) from 2002 to 2014. OXVASC is an ongoing population-based study of the incidence and outcome of all acute vascular events in a population of 92 728 individuals, registered with 100 general practitioners in 9 general practices in Oxfordshire, United Kingdom. The multiple overlapping methods used to achieve near complete ascertainment of all individuals with TIA and ischemic stroke have been reported previously.^15^ Briefly, these included (1) a daily, rapid-access TIA, and stroke clinic to which participating general practitioners and the local emergency department team referred individuals with suspected TIA or minor stroke; (2) daily searches of admissions to medical, stroke, neurology, and other relevant wards; (3) daily searches of the local emergency department attendance register; (4) daily searches of in-hospital death records via the bereavement office; (5) monthly searches of all death certificates and coroner’s reports for out-of-hospital deaths; (6) monthly searches of general practitioner diagnostic coding and hospital discharge codes; and (7) monthly searches of all brain and vascular imaging referrals.

Demographic data, risk factors for atherosclerosis (eg, hypertension, diabetes mellitus, history of smoking, hypercholesterolemia), and history of vascular disease in other vascular beds (symptomatic coronary and peripheral vascular disease) were collected from face-to-face interview and cross-referenced with primary care records.

Patients were considered to have concurrent coronary heart disease if they had at least one of the following conditions: previous myocardial infarction; unstable angina; angina; and history of percutaneous coronary intervention or coronary artery bypass graft surgery. Concurrent symptomatic peripheral vascular disease was defined as having at least one of the following conditions: previous aortic aneurysm rupture; aortic dissection; acute limb ischemia; critical limb ischemia; acute visceral ischemia; intermittent claudication; previous angioplasty or stenting; peripheral arterial bypass graft or amputation. Patients without a history of symptomatic coronary or peripheral vascular disease were classified as having single-territory disease (TIA/stroke only), whereas patients with concurrent coronary or peripheral vascular disease were classified as having double-territory disease and patients with diseases in both coronary and peripheral vascular beds were classified as having triple-territory symptomatic vascular disease.

All patients routinely had brain imaging (computed tomography or magnetic resonance imaging), intracranial and extracranial vascular imaging (carotid Doppler/CTA/MRA/DSA), 12-lead electrocardiography, and routine bloods (ie, full blood count, clotting, C-reactive protein, erythrocyte sedimentation rate, liver function, renal function, thyroid function, electrolytes, and lipid profile) after the event. Echocardiography, 24-hour electrocardiography, and 5-day electrocardiography event recorder (R test) were also done when clinically indicated. Standard secondary preventive treatment was continued or started on the day of the initial clinical assessment, which usually included antithrombotic treatment, antihypertensive drugs, and a statin. Notably, although we routinely prescribed a statin, the exact regime continued for long-term use (ranging from simvastatin 40 mg daily to atorvastatin 80 mg daily) was left to the patient’s primary care physician, who has a responsibility in the UK healthcare system for long-term management of patients.

Patients were followed-up face-to-face at 1, 6, 12, 60, and 120 months by a study nurse or physician to identify any recurrent stroke and other acute vascular events (myocardial infarction, peripheral vascular event), supplemented by review of primary care records. Patients who had moved out of the study area were followed-up via telephone at the same time points as face-to-face follow-up. We recorded all deaths during follow-up with the underlying causes by direct follow-up, via primary care records, and by centralized registration with Office for National Statistics. All recurrent events that occurred during follow-up would also be identified by the ongoing daily case ascertainment.

### Statistical Analysis

Baseline characteristics were compared between all 3 groups (single-territory versus double-territory versus triple-territory), using χ^2^ test for categorical variables and 1-way ANOVA test for continuous variables.

We compared the prevalence of atherosclerotic risk factors (overall number and individual risk factors) and also prevalence of asymptomatic carotid disease (based on vascular imaging performed routinely for all patients as part of the diagnostic workup) in patients with single- versus multiple-territory disease using χ^2^ test and logistic regression analysis adjusted for age and sex.

Kaplan-Meier survival analysis was used to calculate the 1-year, 5-year, and 10-year risks of vascular events during follow-up, censored at death or September 30, 2014, for single-, double-, and triple-territory disease. We compared the following outcomes in patients with single- versus multiple-territory disease using Cox-regression analysis adjusted for age and sex: first major cardiovascular event (any recurrent ischemic stroke, myocardial infarction, acute peripheral vascular event, or vascular death), vascular death, first recurrent ischemic stroke, and first nonstroke acute vascular event (myocardial infarction, acute peripheral vascular event, or sudden cardiac death). Exploratory analyses were performed with additional adjustment for other known vascular risk factors.

Sensitivity analyses were also performed confined to patients with large artery disease according to TOAST classification (Trial of ORG 10172 in Acute Stroke Treatment), excluding patients with known atrial fibrillation at baseline and stratified by the type of the index event (TIA versus ischemic stroke).

All analyses were done using SPSS version 22.

### Standard Protocol Approvals, Registrations, and Patient Consents

Written informed consent or assent from relatives was obtained in all participants. OXVASC was approved by the local research ethics committee (OREC A: 05/Q1604/70).

## Results

Of 2554 patients with a first-in-the-study-period event (1606 ischemic stroke and 948 TIA), 1842 (72.1%) had single-territory disease (TIA/ischemic stroke only), 608 (23.8%) had double-territory, and 104 (4.1%) had triple-territory symptomatic vascular disease.

As shown in Table 1, patients with double- or triple-territory disease were more likely to be on preventative agents for vascular disease before the index TIA/stroke. The proportion of patients on secondary prevention further increased after the index event for all patients (Table 1) but was more intensive in patients with multiple-territory disease (Table 1). At 1-month follow-up, 84 (95.5%) of the 88 patients with triple-territory disease were on antithrombotic agents, 74 (84.1%) on antihypertensive treatment, and 73 (83.0%) were on statins (Table 1). At 1-year follow-up, 72 (97.3%) of the 74 patients with triple-territory disease remained on antithrombotic agents, 63 (85.1%) on antihypertensive treatment, and 61 (82.4%) were still on statins (Table 1).

**Table 1.**
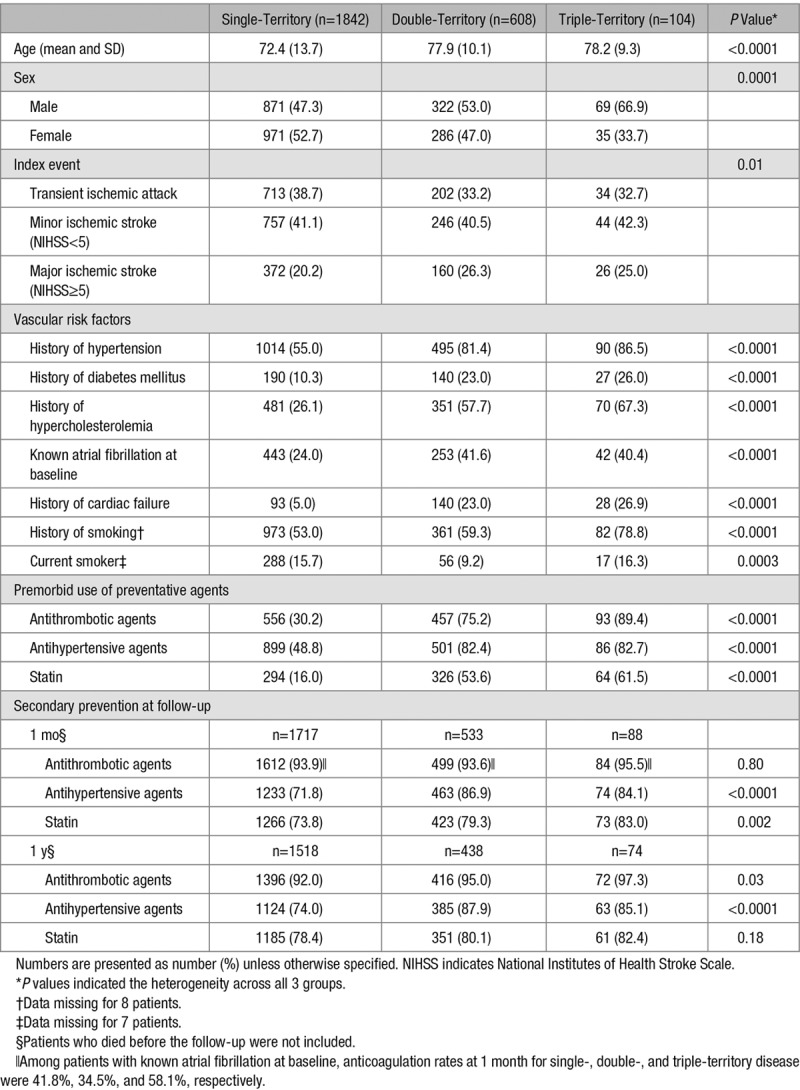
Demographics, Risk Factors, and Secondary Prevention Treatment in Patients With Baseline Single-, Double-, and Triple-Territory Diseases

**Figure 1.**
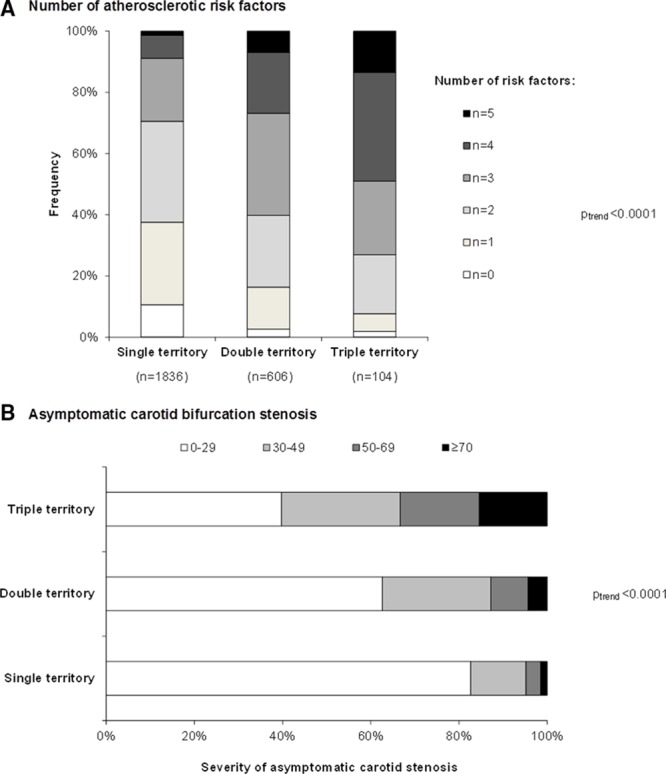
Distribution of numbers of atherosclerotic risk factors (**A**) and severity of asymptomatic carotid bifurcation stenosis (**B**) in patients with single-, double-, and triple-territory disease at baseline. History of smoking data missing for 8 patients in **A**.

As shown in Figure 1, the number of affected vascular beds increased with the numbers of atherosclerotic risk factors (*P*_trend_<0.0001), with the highest prevalence in patients with triple-territory disease (Table 1). Compared with patients with TIA/stroke only, those with double- or triple-territory disease had more hypertension (age/sex-adjusted odds ratio [OR], 3.43; 95% confidence interval [CI], 2.76–4.27; *P*<0.0001; Table 2), diabetes mellitus (OR, 2.89; 95% CI, 2.27–3.66; *P*<0.0001; Table 2), hypercholesterolemia (OR, 4.67; 95% CI, 3.85–5.66; *P*<0.0001; Table 2), and history of smoking (OR, 1.52; 95% CI, 1.26–1.84; *P*<0.0001; Table 2). The same was observed when comparing patients with triple-territory disease to patients with TIA/stroke alone (Table 2) and triple-territory disease was particularly strongly associated with known hypercholesterolemia (OR, 6.80; 95% CI, 4.39–10.53; *P*<0.0001; Table 2), with a baseline mean/SD total cholesterol of 4.5/1.2 mmol/L despite 62% being on statin treatment before the index TIA/stroke (Table 1).

**Table 2.**
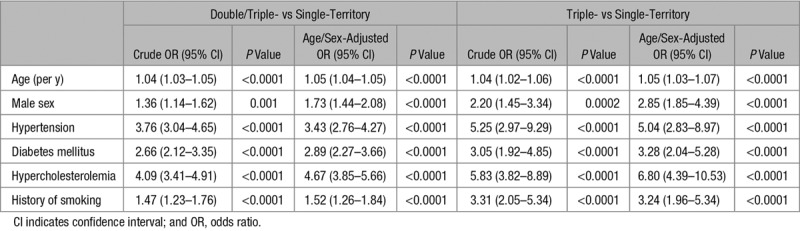
Crude and Age/Sex-Adjusted ORs of Different Atherosclerotic Risk Factors in Multiple-Territory vs Single-Territory Events

Not only did the prevalence of vascular risk factors increase with the number of affected vascular beds, but patients with multiple-territory disease also had more severe asymptomatic carotid bifurcation stenosis (Figure 1). Twenty-six (33.3%) of 78 patients in the triple-territory group had at least 50% asymptomatic stenosis, compared with 69 (4.8%) of 1441 patients in the TIA/stroke only group (age/sex-adjusted OR, 7.39; 95% CI, 4.26–12.81; *P*<0.0001).

**Figure 2.**
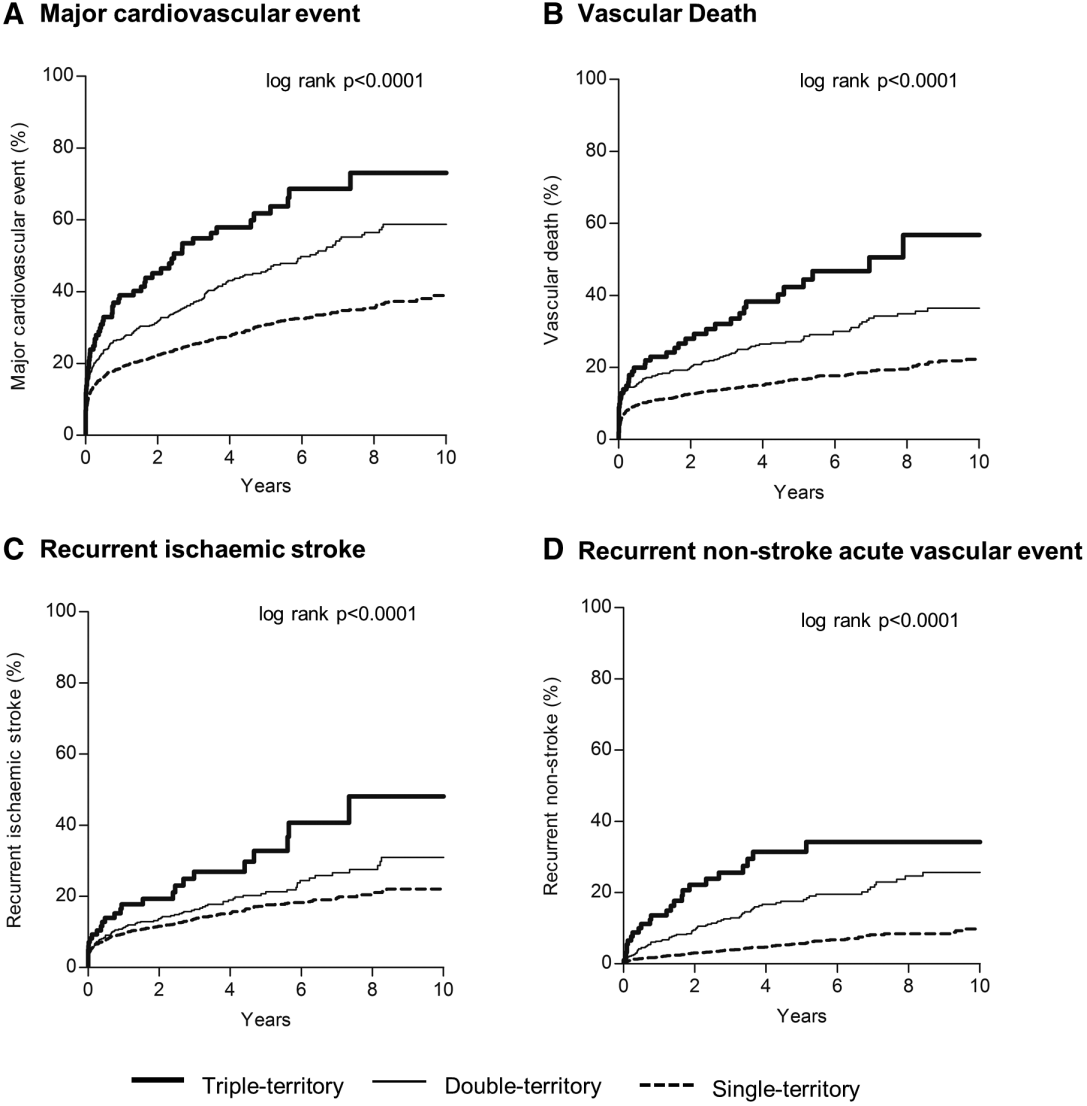
Ten-year risks of recurrent vascular events in patients with baseline single, double, and triple-territory diseases. Panels are for different outcomes. **A**, Major cardiovascular event: any recurrent ischemic stroke, myocardial infarction, acute peripheral vascular event, or vascular death; **B**, vascular death; **C**, recurrent ischaemic stroke; **D**, recurrent nonstroke acute vascular event: myocardial infarction, acute peripheral vascular event, or sudden cardiac death.

**Figure 3.**
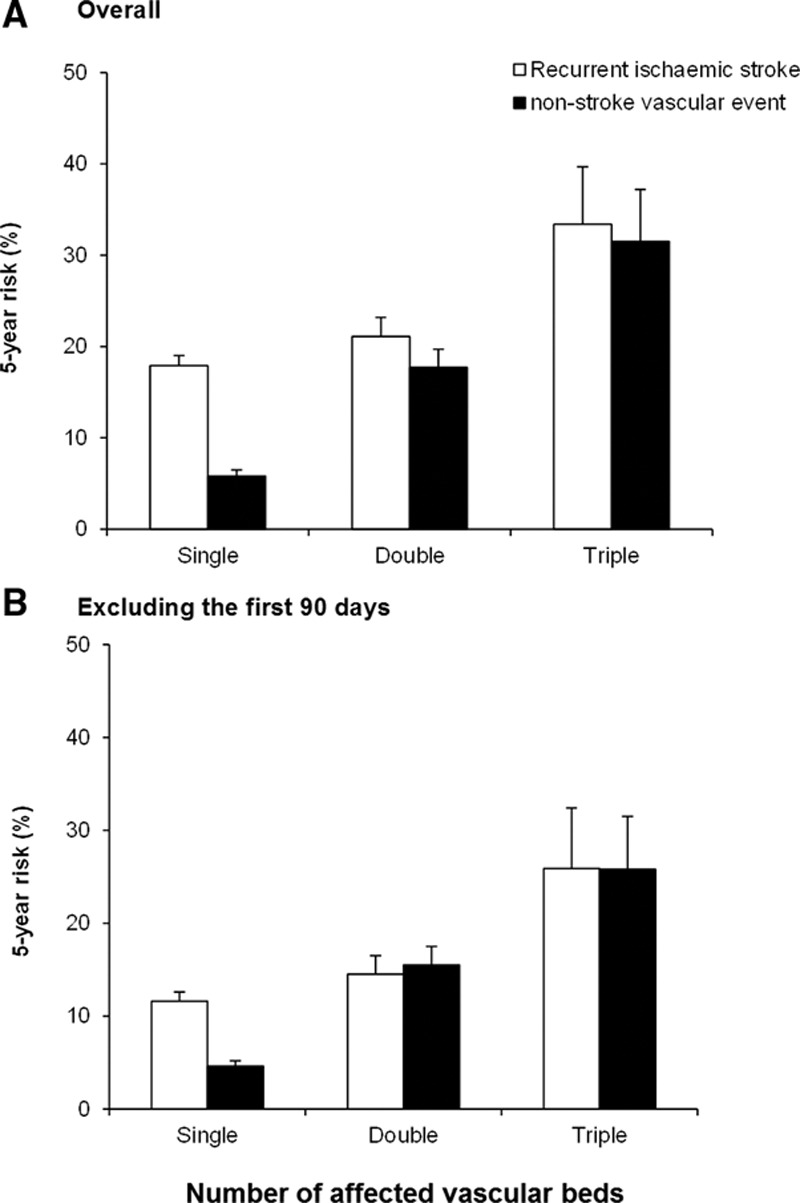
Five-year risk of recurrent ischemic stroke and nonstroke acute vascular event by the number of affected vascular beds. Overall risks (**A**) and risks excluding the first 90-days (**B**) are presented in panels. Error bar represents SE.

During 10 679 patient-years of follow-up, there were 515 vascular deaths, 417 recurrent ischemic strokes, and 203 recurrent nonstroke acute vascular events (136 acute coronary events and 67 acute peripheral events). Despite more intensive secondary prevention in patients with multiple-territory disease, the 5-year risks of major cardiovascular event, vascular death, recurrent ischemic stroke, or recurrent nonstroke acute vascular events increased steeply with the number of territories affected (Figure 2; Figure I in the online-only Data Supplement). Although the risks were particularly front-loading (Figure 2), patients with multiple-territory disease also had higher post 90-day long-term risks of recurrent vascular events (double/triple versus single 10-year major cardiovascular events: 50.7% versus 29.0%; age/sex-adjusted hazard ratio [HR], 1.67; 95% CI, 1.37–2.01; *P*<0.0001; Table 3). The risks were higher in patients with TIA/stroke plus peripheral vascular disease than in patients with TIA/stroke plus coronary artery disease (10-year major cardiovascular events: 60.8% versus 46.1%; age/sex-adjusted HR, 1.58, 95% CI, 1.03–2.43; *P*=0.04), and were highest in those with triple-territory disease (triple versus single major cardiovascular events: 64.2% versus 29.0%; age/sex-adjusted HR, 2.68; 95% CI, 1.86–3.86; *P*<0.0001; Table 3). Moreover, compared with patients with TIA/stroke only, patients with triple-territory disease also had a 2-fold increase of recurrent ischemic stroke (10-year age/sex-adjusted HR, 2.32; 95% CI, 1.39–3.88; *P*=0.001; Table 3), and a 5-fold increase of recurrent nonstroke acute vascular events (HR, 4.62; 95% CI, 2.68–7.98; *P*<0.0001), with the risks of recurrent nonstroke acute vascular events approaching the risks of recurrent ischemic stroke (Figure 3).

**Table 3.**
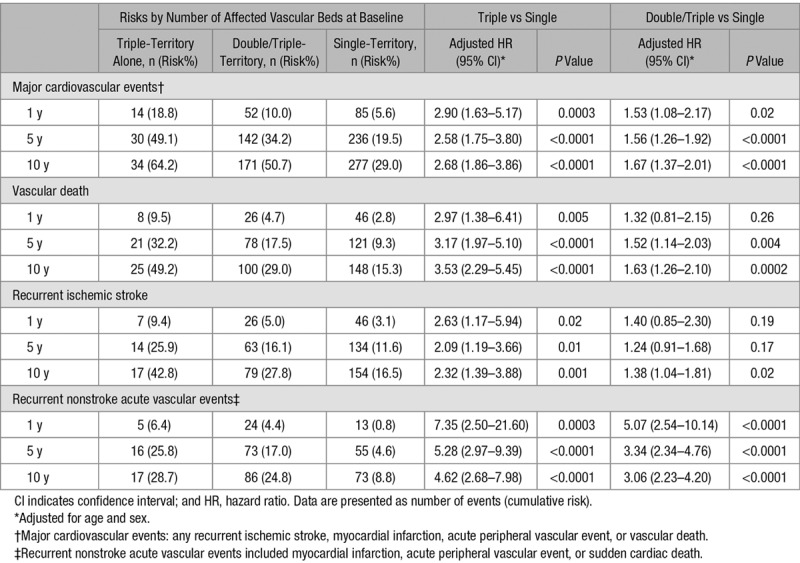
Post 90-Day Cumulative Risks of Vascular Death, Recurrent Ischemic Stroke, or Recurrent Nonstroke Acute Vascular Event Stratified by Number of Affected Vascular Beds at Baseline

Sensitivity analysis confined to patients with large artery disease (Table I in the online-only Data Supplement), excluding patients with known atrial fibrillation at baseline (Table II in the online-only Data Supplement), or stratified by the type of the index event (Table III in the online-only Data Supplement) also showed consistent results. Exploratory multivariate analyses adjusting for other vascular risk factors also suggested that multiple-territory disease was associated with post 90-day long-term risks of recurrent cardiovascular events independent of age, male sex, history of hypertension, diabetes mellitus, hypercholesterolemia, atrial fibrillation, cardiac failure, and history of smoking (Tables IV and V in the online-only Data Supplement).

## Discussion

In this population-based study, we showed that over a quarter of patients presenting with TIA or ischemic stroke also had known symptomatic disease in other vascular beds. As expected, the number of affected vascular beds increased with the numbers of atherosclerotic risk factors. Despite intensive secondary prevention, 10-year risks of recurrent vascular events increased steeply with the number of territories affected. Of particular note, the long-term risks of recurrent nonstroke acute vascular events approached the risks of recurrent ischemic stroke in patients with multiple-territory disease.

Our findings are in line with previous studies showing that despite standard secondary prevention, patients with multiple-territory disease still had a ≈1.5-fold increase of recurrent vascular events or vascular death than patients with TIA or ischemic stroke alone.^13,14,16–18^ However, our estimates of the absolute risks were much higher than previous studies, even after excluding the acute phase post-TIA/ischemic stroke.^13,17^ For example, the 90-day to 1-year vascular death in patients with multiple-territory disease was 4.7% in OXVASC versus 2.8% in the REACH registry (Reduction of Atherothrombosis for Continued Health),^17^ and the risk of all major vascular events was 8.6 per 100 patient-years versus 5.0 in the SMART study (Second Manifestations of Arterial Disease).^13^ These differences probably reflect the larger number of elderly patients with multiple comorbidities in OXVASC owing to the population-based design and the longer period of follow-up.

That the number of affected territories still predicts a poor outcome in a cohort on current standard secondary prevention highlights the unmet need for more effective treatment in TIA or ischemic stroke patients with symptomatic disease in multiple vascular beds. We found that the number of atherosclerotic risk factors increased with the number of affected vascular beds, with particularly strong associations with hyperlipidemia, reflecting the importance of lipids and smoking in coronary and peripheral vascular disease.^19,20^ Both the recent FOURIER (Further Cardiovascular Outcomes Research With PCSK9 Inhibition in Subjects With Elevated Risk) and the REVEAL (Randomized Evaluation of the Effects of Anacetrapib Through Lipid Modification) trials showed that in patients with atherosclerotic cardiovascular disease there is some additional benefit from lowering of cholesterol levels below current targets.^11,12^ Although our patients with multiple-territory disease were usually on premorbid statins, they still had total cholesterol of 4.5 mmol/L at baseline, and although lipid-lowering was intensified thereafter in the majority, additional treatments might be justified.^21,22^ Moreover, previous studies have shown that there is a systemic predisposition to atherosclerosis,^23–26^ and we found that in patients with multiple-territory disease, the long-term risks of nonstroke acute vascular events approached the risks of recurrent ischemic stroke.

In our exploratory analyses, we found that numbers of affected vascular beds were associated with long-term risks of recurrent major cardiovascular events independent of known vascular risk factors, and multiple-territory disease seemed to be a stronger predictor than vascular risk factors measured at baseline. This perhaps reflects the fact that crude prevalence of reported vascular risk factors is not always an adequate measure of risk because of measurement error, premorbid preventative treatment, and different individual susceptibility. Hence, the number of affected vascular beds is a perhaps more informative summative measure of risk.

Although we consider our conclusions to be valid, our study has limitations. First, we did not screen patients for asymptomatic coronary or peripheral vascular disease and will have underestimated the real burden of multiple-territory disease. However, screening for asymptomatic coronary or peripheral vascular disease is not routine in clinical practice. Moreover, even in patients with no known coronary heart disease, statins have been shown to reduce major coronary-related events in patients with TIA or ischemic stroke.^6^ Second, although the majority of the patients were on a statin during long-term follow-up, the exact regime varied (usually ranging from simvastatin 40 mg daily to atorvastatin 80 mg daily). However, this heterogeneity does reflect real-world clinical practice. Third, we did not routinely recheck lipid levels during follow-up, as this is the responsibility of primary care physicians in the UK healthcare system, and so we did not have systematic data on the quality of cholesterol control. Fourth, although we used several overlapping methods (ie, interviews, ongoing daily ascertainment, primary diagnostic coding, death certificates, and national hospital coding) to achieve near complete follow-up, a small proportion of patients (<1%) emigrated from the United Kingdom and could not always be followed-up. Finally, our results based on a predominantly white population in the United Kingdom might not be generalizable to other countries.

To conclude, in a population-based cohort of TIA and ischemic stroke patients treated with contemporary standard secondary prevention, we found that patients with multiple-territory disease had a very high risk of recurrent vascular events during long-term follow-up, suggesting that number of affected vascular beds could potentially be a simple clinical rule in identifying patients who are at high risk of recurrent vascular events. Our updated risk estimates with contemporary secondary prevention therapies could also help to inform the design of future randomized trials. Finally, patients with the multiple-territory disease might benefit from more intensive prevention with novel therapies and should be the focus of future clinical trials.

## Acknowledgments

This article is dedicated to Rose Wharton, who provided statistical support but sadly died prior to publication. We are also grateful to all the staff in the general practices that collaborated in the Oxford Vascular Study: Abingdon Surgery, Stert St, Abingdon; Malthouse Surgery, Abingdon; Marcham Road Family Health Centre, Abingdon; The Health Centre, Berinsfield; Key Medical Practice; Kidlington; 19 Beaumont St, Oxford; East Oxford Health Centre, Oxford; Church Street Practice, Wantage. We also acknowledge the use of the facilities of the Acute Vascular Imaging Centre, Oxford.

## Sources of Funding

Wellcome Trust, Wolfson Foundation, British Heart Foundation, and the National Institute for Health Research (NIHR) Oxford Biomedical Research Centre. Dr Rothwell is in receipt of an NIHR Senior Investigator Award and Dr Heldner is in receipt of a Swiss National Science Foundation Advanced Postdoc Mobility Grant, of a Novartis Foundation for medical-biological Research Grant and of a B. Braun Foundation Grant. Drs Heldner and Li collected data, did the statistical analysis and interpretation, wrote and revised the article. Drs Lovett, Kubiak, and Lyons collected the data. Professor P.M. Rothwell conceived and designed the overall study, provided study supervision and funding, acquired, analyzed and interpreted data, and wrote and revised the article. The views expressed are those of the author(s) and not necessarily those of the NHS, the NIHR, or the Department of Health.

## Disclosures

None.

## Supplementary Material
