# Supplementary material for: Long-Term Prognosis of Patients With Transient Ischemic Attack or Stroke and Symptomatic Vascular Disease in Multiple Arterial Beds
Source: Stroke. 2018 Jun 7;49(7):1639–46. doi: 10.1161/STROKEAHA.118.020913 (PMC6023586; doi:10.1161/STROKEAHA.118.020913)
Supplement: Supplementary file 1 [file str-49-1639-s001.pdf]

SUPPLEMENTAL MATERIAL

Figure I 10-year risks of myocardial infarction/sudden cardiac death and acute peripheral vascular events in patients with baseline single, double and triple-territory diseases

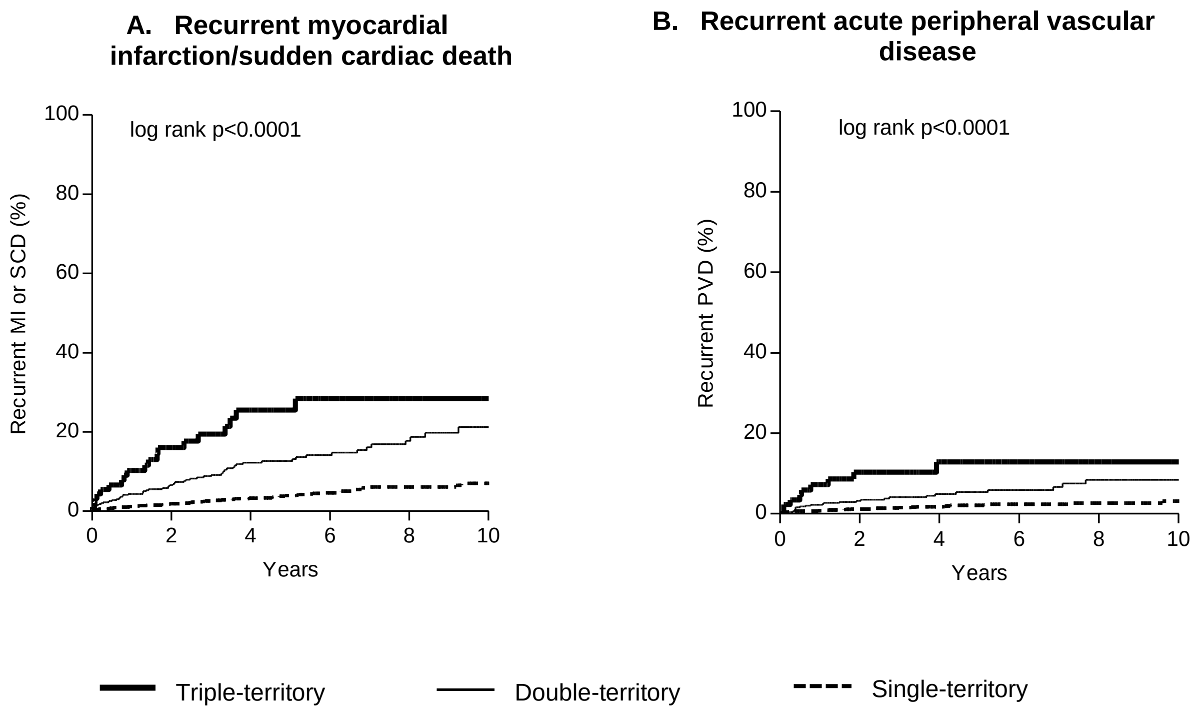

Table I Post 90-day 5-year risks of death and recurrent vascular events in patients with index TIA or ischaemic stroke stratified by number of affected vascular beds at baseline in those with large artery disease only

	**Number of affected vascular beds at baseline:**			**Triple vs. single**		**Double/triple vs. single**	
	Triple-territory n (risk%)	Double/triple-territory n (risk%)	Single-territory n (risk%)	Adjusted HR† (95%CI)	p	Adjusted HR† (95%CI)	p
Major cardiovascular events‡	8 (42.4)	12 (30.1)	23 (17.5)	2.63 (1.16-5.98)	0.02	1.67 (0.91-3.06)	0.10
Vascular death	3 (16.0)	7 (10.2)	5 (3.4)	3.55 (0.82-15.41)	0.09	2.32 (0.73-7.41)	0.16
Ischaemic stroke	4 (20.7)	9 (15.1)	13 (10.7)	2.24 (0.72-6.95)	0.16	1.24 (0.53-2.93)	0.62
Non-stroke events	5 (25.8)	15 (24.0)	11 (8.0)	3.24 (1.07-9.78)	0.04	2.61 (1.18-5.77)	0.02

Data are presented as number of events (cumulative risk). † adjusted for age and sex. ‡Major cardiovascular events: any recurrent ischaemic stroke, myocardial infarction, acute peripheral vascular event or vascular death.

Table II Risks of death and recurrent vascular events in patients with index TIA or ischaemic stroke stratified by number of affected vascular beds at baseline, excluding patients with known atrial fibrillation at baseline

	**Number of affected vascular beds at baseline:**			**Triple vs. single**		**Double/triple vs. single**	
	Triple-territory n (risk%)	Double/triple-territory n (risk%)	Single-territory n (risk%)	Adjusted HR† (95%CI)	p	Adjusted HR† (95%CI)	p
**Post 90-day to 5-year**							
Major cardiovascular events‡	20 (46.5)	82 (31.6)	169 (17.5)	2.75 (1.72-4.41)	<0.0001	1.61 (1.23-2.10)	0.001
Vascular death	11 (24.9)	35 (12.7)	73 (7.2)	2.84 (1.49-5.41)	0.002	1.37 (0.92-2.06)	0.13
Ischaemic stroke	11 (27.2)	40 (16.0)	97 (10.3)	2.62 (1.39-4.94)	0.003	1.39 (0.96-2.03)	0.08
Non-stroke events	12 (26.3)	49 (17.3)	47 (4.9)	4.84 (2.53-9.26)	<0.0001	3.17 (2.11-4.75)	<0.0001
**Post 90-day to 10-year**							
Major cardiovascular events‡	23 (62.2)	99 (46.0)	202 (26.9)	2.87 (1.85-4.45)	<0.0001	1.67 (1.31-2.13)	<0.0001
Vascular death	15 (47.5)	50 (24.5)	91 (12.2)	3.73 (2.14-6.51)	<0.0001	1.62 (1.15-2.29)	0.006
Ischaemic stroke	13 (42.4)	48 (24.3)	113 (14.9)	2.79 (1.56-5.02)	0.001	1.44 (1.02-2.04)	0.036
Non-stroke events	13 (30.0)	59 (25.9)	64 (9.5)	4.25 (2.31-7.83)	<0.0001	2.87 (2.00-4.10)	<0.0001

Data are presented as number of events (cumulative risk). † adjusted for age and sex. ‡Major cardiovascular events: any recurrent ischaemic stroke, myocardial infarction, acute peripheral vascular event or vascular death.

Table III Post 90-day 5-year risks of death and recurrent vascular events in patients with index TIA or ischaemic stroke stratified by number of affected vascular beds at baseline

	Number of affected vascular beds at baseline:			Triple vs. single		Double/triple vs. single	
	Triple-territory n (risk%)	Double/triple-territory n (risk%)	Single-territory n (risk%)	Adjusted HR† (95%CI)	p	Adjusted HR† (95%CI)	p
**Ischaemic stroke**							
Major cardiovascular events‡	18 (44.9)	95 (36.9)	179 (25.5)	1.91 (1.16-3.14)	0.01	1.37 (1.06-1.76)	0.02
Vascular death	10 (24.1)	45 (16.3)	91 (11.7)	2.14 (1.08-4.22)	0.03	1.20 (0.84-1.72)	0.31
Ischaemic stroke	9 (25.7)	47 (19.6)	104 (15.8)	1.66 (0.83-3.32)	0.16	1.18 (0.83-1.68)	0.35
Non-stroke events	9 (22.2)	44 (16.5)	35 (5.1)	4.45 (2.07-9.59)	0.0001	3.12 (1.99-4.89)	<0.0001
**TIA**							
Major cardiovascular events‡	12 (58.9)	47 (29.8)	57 (11.4)	4.86 (2.59-9.12)	<0.0001	2.07 (1.40-3.06)	0.0003
Vascular death	11 (44.7)	33 (19.1)	30 (5.9)	6.92 (3.45-13.89)	<0.0001	2.52 (1.53-4.15)	0.0003
Ischaemic stroke	5 (28.3)	16 (10.4)	30 (6.2)	3.73 (1.44-9.66)	0.007	1.31 (0.71-2.42)	0.39
Non-stroke events	7 (32.3)	29 (17.8)	20 (4.0)	7.06 (2.93-17.04)	<0.0001	3.72 (2.08-6.66)	<0.0001

Data are presented as number of events (cumulative risk). † adjusted for age and sex. ‡Major cardiovascular events: any recurrent ischaemic stroke, myocardial infarction, acute peripheral vascular event or vascular death.

Table IV Exploratory analyses of the associations of multi-territory disease and post 90-day to 5-year risks of recurrent major cardiovascular events adjusting for vascular risk factors

	HR (95%CI)	p
Tripe- vs. single-territory	2.01 (1.33-3.04)	0.001
Age	1.06 (1.04-1.07)	<0.0001
Male sex	0.93 (0.72-1.22)	0.61
Hypertension	1.25 (0.95-1.65)	0.11
Diabetes	1.30 (0.92-1.84)	0.13
Hypercholesterolaemia	1.15 (0.88-1.52)	0.31
Atrial fibrillation	1.38 (1.05-1.81)	0.02
Heart failure	1.17 (0.74-1.83)	0.50
History of smoking	1.30 (1.00-1.69)	0.049
	**HR (95%CI)**	**p**
Tripe/double- vs. single-territory	1.31 (1.04-1.66)	0.02
Age	1.05 (1.04-1.07)	<0.0001
Male sex	1.00 (0.81-1.25)	0.97
Hypertension	1.28 (1.00-1.63)	0.049
Diabetes	1.28 (0.96-1.69)	0.09
Hypercholesterolaemia	1.08 (0.86-1.35)	0.52
Atrial fibrillation	1.36 (1.09-1.71)	0.006
Heart failure	1.04 (0.75-1.45)	0.80
History of smoking	1.26 (1.01-1.56)	0.04

Table V Exploratory analyses of the associations of multi-territory disease and post 90-day to 5-year risks of recurrent ischaemic stroke and recurrent non-stroke acute vascular events adjusting for vascular risk factors

A. Recurrent ischaemic stroke

	**HR (95%CI)**	**p**
Tripe- vs. single-territory	1.75 (0.97-3.15)	0.06
Age	1.04 (1.02-1.05)	<0.0001
Male sex	1.00 (0.71-1.42)	1.00
Hypertension	1.37 (0.95-1.99)	0.09
Diabetes	1.31 (0.83-2.08)	0.25
Hypercholesterolaemia	1.23 (0.86-1.76)	0.27
Atrial fibrillation	1.45 (1.00-2.11)	0.05
Heart failure	0.64 (0.29-1.41)	0.27
History of smoking	0.99 (0.70-1.39)	0.93
	**HR (95%CI)**	**p**
Tripe/double- vs. single-territory	1.08 (0.77-1.50)	0.65
Age	1.04 (1.02-1.05)	<0.0001
Male sex	1.02 (0.75-1.37)	0.92
Hypertension	1.26 (0.90-1.75)	0.17
Diabetes	1.25 (0.84-1.84)	0.27
Hypercholesterolaemia	1.25 (0.91-1.70)	0.16
Atrial fibrillation	1.38 (1.01-1.90)	0.04
Heart failure	0.58 (0.32-1.04)	0.07
History of smoking	1.02 (0.76-1.37)	0.92

B. Recurrent non-stroke acute vascular events

	**HR (95%CI)**	**p**
Tripe- vs. single-territory	3.60 (1.91-6.77)	<0.0001
Age	1.07 (1.04-1.09)	<0.0001
Male sex	1.14 (0.68-1.92)	0.63
Hypertension	1.80 (0.98-3.30)	0.06
Diabetes	1.63 (0.90-2.97)	0.11
Hypercholesterolaemia	1.23 (0.73-2.09)	0.43
Atrial fibrillation	0.60 (0.32-1.14)	0.12
Heart failure	1.26 (0.55-2.90)	0.58
History of smoking	1.80 (1.04-3.12)	0.04
	**HR (95%CI)**	**p**
Tripe/double- vs. single-territory	2.80 (1.88-4.16)	<0.0001
Age	1.06 (1.04-1.08)	<0.0001
Male sex	1.31 (0.89-1.91)	0.17
Hypertension	1.65 (1.02-2.66)	0.04
Diabetes	1.74 (1.14-2.66)	0.01
Hypercholesterolaemia	1.03 (0.70-1.53)	0.87
Atrial fibrillation	0.77 (0.51-1.17)	0.22
Heart failure	1.03 (0.61-1.75)	0.91
History of smoking	1.54 (1.04-2.29)	0.03
